# A qualitative study on diverse experiences of medication safety among foreign-born persons living in Sweden

**DOI:** 10.1186/s12889-024-18711-7

**Published:** 2024-05-07

**Authors:** Lisa Hultin, Ulrika Pöder, Mariann Hedström, Anna Ekman, Katarina Hjelm

**Affiliations:** 1 Department of Public Health and Caring Sciences, Uppsala University, IFV, Husargatan 3, Box 564, Uppsala, 751 23 Sweden; 2 Primary Care and Health, Uppsala County Council, Uppsala, Sweden

**Keywords:** Communication barriers, Medication safety, Migrants, Patient safety, Primary health care, Qualitative research, Self care

## Abstract

**Background:**

The ongoing global migration has led to multicultural societies, with many migrants who do not speak the official language in the host country. This could contribute to communication problems with staff in healthcare and a risk to patient safety. Research on patient safety in medication use in migrants is an under-researched area. The aim was to explore diverse foreign-born people’s experiences and perceptions of self-management of medication and determine if home-based practice patterns have implications on medication safety, and what factors may support safe medication use.

**Methods:**

A qualitative explorative study, with individual semi-structured interviews and participant observations in the patients’ home. Qualitative content analysis was applied.

**Results:**

A purposeful sample of 15 foreign-born persons identified by healthcare staff as having language difficulties in Swedish that may pose a safety risk in connection with medication use at home, was selected. Three categories were identified. The first category showed respondents being dependent on another person, having experiences of not receiving information about their medications due to language barriers, having difficulties getting access to the healthcare centre and feeling distrusted/misunderstood. The second category showed respondents being independent and self-motivated Although they struggled, they managed to get access/contact with the healthcare centre themselves and felt understood/listened to by the staff. The last category concerned factors that facilitating medication use; such as having a medication list in the respondents’ own language and offering a choice of language on the answering machine at the healthcare centre. Although they knew it was impossible to get an interpreter at the pharmacy, they felt safe knowing there was always a solution for receiving understandable information.

**Conclusion:**

The findings highlighted that language barriers can complicate the communication between migrants and the healthcare, which can affect the medication safety. Understanding of this group is essential to improve the cooperation between patients and staff, leading to culturally congruent care. This knowledge should be used in healthcare to understand the gap in communication to increase patient safety. Further research from other angles, e.g. pharmacy/healthcare staff and relatives is needed to identify and evaluate facilitation to improve the outcome of the intended medication treatment.

## Background

Migration is increasing worldwide. Approximately 281 million persons are migrants globally, and the number is predicted to increase in the future [1]. With this ongoing global migration and globalisation, many societies have become multicultural, with many migrants (immigrants and refugees) who do not speak the official language of the host country. This could contribute to a communication problem with the healthcare system, inappropriate treatment, and insufficient medication safety [2–4]. Good communication is essential to ensure patient safety, in terms of use of medication by patients [5]. A recent systematic review [6] on safety of healthcare for ethnic minority patients (some migrant groups included) concluded that there has been limited focus on improving safety for ethnic minority populations, which remains an under-researched area. People of ethnic minority backgrounds and migrants might experience inequity in the safety of care and are at a higher risk of patient safety incidents and have, for example, higher rates of adverse drug events and dosing errors compared to the population of origin [6]. Another review of ethnic minority patients (limited number of migrants) in the UK aimed to establish types of and possible medication-related problems experienced [7]: cultural and religious beliefs, different expectations, language and communication barriers, lack of knowledge of the healthcare services, and underestimating patients’ desire for information were discussed as factors related to problems of taking medications as advised, concern for side effects, risk of adverse drug reactions, and problems accessing healthcare services. However, only two out of fifteen studies included were performed in the patients’ home, and none had made observations of patient’s self-administration. It was concluded that further research is needed and designed to identify and address the needs and perspectives of these groups, particularly as little evidence exists on what influences medication-related problems among migrants.

For migrants, it can be difficult to navigate in, and to get access to, healthcare, within the often complicated and exchangeable systems and models in the organisation of healthcare [3, 4]. This can be explained by language difficulties but also cultural dissimilarities in beliefs about health and illness and knowledge about the existing healthcare organisation [4, 8–10]. Lack of social support and other socioeconomic factors might contribute to migrants having more difficulties in searching for care [4]. In general, migrants have poorer health and shorter life expectancy than the population of origin [11, 12].

In Sweden, the number of migrants has increased from 11% of the registered population in 2000 to approximately 20% in 2022 [13]. People who have migrated to Sweden come from a variety of countries, at different times, and with different reasons for migration. The pattern of migration changed in the mid 1980s into non-European refugees (mainly from the Middle East and Africa) from labour migrants from Scandinavian and European countries dominating during the industrialisation from the post-war era. In 2015, an unusually high number of migrants from Afghanistan, North Africa, and Syria applied for asylum to nearly all European countries. The third largest recipient country was Sweden [14]. The foreign-born population is therefore characterised by great diversity. The group of persons born outside Europe who have spent 0–9 years in Sweden are characterised largely by the refugee migration that took place in recent years. However, many migrants have spent a long time in Sweden [15].

Using medications is a complex process, and language difficulties can play a role in the patient’s adherence to prescribed medications [16]. Transcultural care emphasises the need to provide care based on the individual’s or group’s culturalhealth beliefs, practice, and values. This determines health-related behaviour, including self-management of medications, and thus affects health. Thus, the primary goal of healthcare should be to provide culturally congruent care. The health care needs to be designed in a partnership between the individual and healthcare staff and an integrated care based on a combination of holistic, generic, and professional care knowledge, and in multi-professional collaboration, to achieve the goal of health and to prevent illness [17].

How migrants experience medication management at home has been studied to a very limited extent; however, no studies have been found that have been performed in patients’ home, including observations on use of and handling of medications. Little evidence exists on what influences medication-related problems among ethnic minorities, particularly migrants, despite increased diversification of populations in countries throughout the world as well as what support is needed to optimise the use of medications [6, 7].

Therefore, this study explores diverse foreign-born people’s experiences and perceptions of self-management of medication and determine if there are implications of home-based practice patterns on medication safety, and what factors may support safe medication use.

## Methods

### Design

A qualitative explorative study design with individual semi-structured interviews and participant observations was used to collect data, as it provides new insights and increases understanding in an area that has previously been investigated to a limited extent. Semi-structured interviews allowed the respondents to discuss the content within a given frame of questions and aimed to reach a nuanced and deeper understanding of the perceptions and experiences of self-management of medications [18]. Semi-structured interviews may be favourable when studying experiences and perceptions. These often take into account gender and cultural variations, whilst acknowledging there is something fundamental regardless of these factors [19]. Participant observations based on an observation scheme facilitate collection of data concerning practical procedures and provide complementary information [18]. The purpose of the observations was to study first-hand what was going on rather than simply assuming what we know. Direct observation of patient care has been used as a reliable qualitative research method to measure errors and adverse events in healthcare [20]. This method provides insight into the unspoken elements and connects the researcher more closely to the basic human experience [18].

### Setting/inclusion sites

Respondents were asked for participation by staff at a primary healthcare centre and a voluntary organisation (a cooperation with the municipality and the church) in an immigration dense area in Sweden. The voluntary organisation organises courses and gatherings for immigrants to promote the integration process.

### Study population and inclusion criteria

A purposeful sampling strategy [18] was applied. Foreign-born people identified by healthcare professionals as having language difficulties in Swedish that may pose a safety risk in connection with medication use at home were invited to participate. Excluded were individuals < 18 years old or having language problems due to mental or somatic disease or known current drug abuse.

Individuals were asked by staff at the healthcare centre and at the voluntary organisation, and they replied by filing in a reply coupon or answered directly to the question; thereafter, a registered nurse (LH) contacted them to set a time and place for the interview. When the person agreed to participate, the medication list from the medical record was collected (with consent from the informant) from the healthcare centre where the patient was registered, as a support when discussing handling of medication.

### Data collection instruments

A semi-structured interview guide and an observation scheme were developed based on a literature review, peer-review with experts, and standardised/validated instruments [21]. Both were based on the patient’s self-reported use of medication, and observation items within domains known to be relevant to safe medication management, including adherence, knowledge, polypharmacy, attitudes, physical and cognitive ability, and lifestyle [22, 23]. The semi-structured interview guide included standardised questions on demographic data and questions on four areas: usage of medication, acquisition of medication, facilitation of medication, and conformity with prescribed medication. Probing questions were posed when appropriate, such as what kind, where, and why. Focus areas in the observation scheme were: how the person takes their medication, preparations of medication, time, hygiene, use of medication list, and support in taking/handling medication.

A pilot study was conducted with two persons to test the interview guide for content, understanding and to check the consistency and the observation scheme. As the interviews and observations turned out well, all the material was included in the study.

### Data collection/process

Data were collected between May 2022 and January 2023.

The interviews and observations were conducted in the respondents’ homes (*n* = 9) or at the voluntary organisation (*n* = 6). A female registered nurse with extensive experiences of caring for patients in home care (LH) collected all the data. She was not involved in the medication management of the study respondents and was not affiliated with the healthcare centre or the voluntary organisation. An authorised interpreter of the same sex and who spoke the same language as the respondent was used.

To enable a comparison between the medication profile and the prescribed medications during the observation and interview with the respondents, the nurse used the respondents’ medication list from the medical record.

The nurse conducted individual interviews and observations of the participants (noted on an observation scheme) in the respondents’ home or in a quiet separate room at the voluntary organisation while the respondent prepared and took their medication. The observation scheme was filled in by the nurse. Occasionally, the nurse asked the respondent for clarification regarding their experiences; otherwise, the observer was quiet. In cases where the observations were conducted at the voluntarily organisation, information regarding the home environment and storage of medications was only requested verbally. The individual interviews were held directly after the observations.

If a discrepancy was noted between the patients’ self-reported use of medication and the medication prescribed, then provided that the respondent consented, a certain contact person (registered nurse) at the healthcare centre was contacted, with the goal of informing the physician responsible for the patient (*n* = 1).

The interviews and the observations lasted between 0.5 and 2 h (median 33 min). The interviews were audio-taped, and field notes that were both descriptive and reflective were taken.

### Data analysis

Data were analysed by inductive, qualitative content analysis to identify recurrent and overarching categories [18]. Interviews were transcribed verbatim by a professional transcription service. The unit of analysis consisted of both interview transcripts and observations schemes. Data collection and analysis proceeded simultaneously, and the analysis was based on openness for variation in the data and a search for patterns, regularities, and contradictions by comparing the statements from the different respondents. Sampling was continued until the researchers agreed that informational redundancy was achieved, that is, the point at which no new information or themes emerge from the data. First, the transcript from the interviews and the notes from observations schemes were read through thoroughly several times to get a sense of the whole. Meaning-bearing units, that signify the parts of sentences/phrases that carry a meaning were identified and coded as close to the text as possible. Thereafter, they were sorted and compared to identify similarities and differences to find patterns; finally, they were grouped into sub-categories and categories.

In order to increase the trustworthiness of the results, investigator triangulation was used to validate the findings [18]. The first author conducted the initial analysis and then discussed the codes with the co-authors in order to compare them and verify their categorisation. If needed, the results were discussed among the researchers until consensus was reached. To increase the credibility, the analysis proceeded to the point when no new information appeared in order to achieve maximum of variation. Careful descriptions of the method are given to ensure dependability. Confirmability was reached by illustrating the content of the categories by illuminating quotations.

## Results

### Characteristics of the study population

The study included 15 foreign-born persons, all except one female, aged between 42 and 72, most with an educational level below primary school (*n* = 8), and reporting no or limited ability to speak Swedish (*n* = 15). All persons were identified by staff as having language difficulties in Swedish posing a safety risk in connection with their medication use at home. Medications treating depression, hypertension, migraine, anxiety and diabetes were commonly used. All but one lived together with their family (Table 1). The respondents were mainly refugees originating from countries in the Middle East and with a median time of residence in Sweden of 11 years (4–46 years) (Table 2).

Approximately half of the respondents reported being dependent on government support for their medication costs (*n* = 8). The others thought the cost of medication was too expensive (*n* = 3) or manageable (*n* = 4), as the Swedish system allows for a maximum yearly medication cost; thereafter, the medication is free. Half of the respondents answered that they were willing to accept a generic medication if needed. Furthermore, almost all the respondents (*n* = 12/15) stated in the interviews that they fasted every year for a month; hence, every day during this period, they do not eat between sunrise and sunset. They either took their medication before sunrise, after sunset, or took half of the medication before sunrise and half after the sunset. If anyone was ill, became ill or too old, they did not need to fast.

**Table 1:** Characteristics of the respondents

**Variable**	
Gender	Women = 14, Men = 1
Age (years) ^1^	66.5(42–72)
Time of residence in Sweden (years) ^1^	11(4–46)
**Educational level**	
No education/illiterate	2
Public school < 6 years	2
Primary school < 9 years	4
Secondary school ≤ 12 years	4
University < 2 years	-
University ≥ 2 years	3
**Reason for migration**	
Refugee	11
Employment	-
Relative	4
**Living conditions**	
Alone	1
Living together with the family	14
**Self-reported fluency in the Swedish language**	
Completely	-
Lack of	4
Limited	7
Not at all	4
**Contacts with healthcare (homecare)**	0
**Medications/Nature cure remedies from home country**	0
**Fasting once a year**	12

^1^ Median (range)

**Table 2:** Country of birth of the study population

**Country**	**n**
Syria	7
Afghanistan	2
Kurdistan	1
Iran	1
Jordan	1
Bangladesh	1
Eritrea	1
Somalia	1

### Categories and sub-categories

Three categories were identified in the analysis of the interviews and observations; “Dependent on another person”, “Independent and self-motivated” and “Factors that can facilitate medication use” (Table 3), (P = quote from individual interview and respondent/person = event from observation, P1 and person 1 correspond to the same person).

**Table 3:** Categories and sub-categories from the analysis

Categories	Sub-categories
Dependent on another person	- Not receiving information about medication because of language problems - Difficulties getting in touch - Feeling distrusted and misunderstood by the healthcare
Independent and self-motivated	- Managing to reach out and contact the healthcare centre - Feeling understood and listened to
Factors that can facilitate medication use	- Possibilities to translation - There’s always a solution at the pharmacy to receive understandable information

### Dependent on another person

This category captures the experiences of the respondents who described not being informed about their medication or not understanding the information given. They thought it was difficult to access the primary healthcare centre and felt misunderstood and distrusted. Thus, they became dependent on support from others in handling their medications.

#### Not receiving information about medication because of language problems

The respondents felt that they did not receive enough or any information from the doctor about their medication or comprehend the information if it was given. They would have liked to receive information about why (the indication), when and how they should take the medication and the potential side effects. Even though the respondents expressed there is always an interpreter or a family member available during the visit at the healthcare centre, they still did not feel that they received this information. This led to them stopping to take the medication, taking incorrect dosage and/or not daring to take medications:

*The doctors said that according to the tests, the dose of Levaxin should be changed, but I don’t even know how they would change it and what it would be. He/she hasn’t said anything since. (P3)*

This was also confirmed during observation of Respondent 1: “The person starts to eat breakfast and explains that he/she always take the medication during breakfast time, shows various boxes with pills and insulin. Shows a box with insulin that is unopened and explains that he/she does not understand why and when to take the insulin, and why it has not been opened. When compared against the medication list, it turns out that it is insulin, with long-term effect that has not been taken due to lack of information”.

Some respondents expressed that they take their medication but without knowing on what indication and some also described being scared to take them because of the lack of information leading to a lack of understanding. Problems were also reported related to not receiving information on test results from blood or x-ray. During the observation of Respondent 3: ‘The person reaches inside a plastic bag in which boxes of medication are stored. Takes out one box with pills and explains that it’s not possible to read on the box or the medication list as it is in Swedish; therefore, it’s difficult to understand when to take them and when to contact the doctor again. The person shrugged his/her shoulders and head, resigned and expressed that “this is impossible to understand”’:

*No, no. No one is helping me with that. I would like to know if there are side effects from the medications, what is it used for…, they have side effects on the kidneys, maybe or the liver or something. But nobody tells me about this. (P12)*

#### Difficulties getting in touch with staff in healthcare

Respondents experienced that it was difficult to get in touch with the healthcare centre due to language problems. They did not understand the Swedish-speaking answering machine when calling the healthcare centre, and they were unable to speak with the nurse at the healthcare centre in case they got through the different choices with the answering machine. They were totally dependent on another person who contacted the centre to make an appointment. Since they could not manage to book an appointment themselves, they were often late with follow-ups, sometimes days, weeks or even more. Some of them expressed that they went to the hospital’s emergency department instead, since it was impossible for them to reach the healthcare centre. During the observation of Respondent 5: ‘The person searches for the correct box when the daughter who is there for the visit helps to find the correct box and explains that her mother got sick and could not reach the healthcare centre to get a prescription for her medication. Thus could not get the correct medication at the pharmacy; therefore, the mother went to the emergency department, since the daughter was out of town and could not help’:

*I got the syringe and the tablets. And then he told me “you have to contact your doctor at the health centre”, but I don’t know how, I can’t do it* (answering machine). *(P5)*

*Yes. Yes, it is always the children who help. Unfortunately, that’s the way it is; roles are reversed in Sweden, so it’s the parents… it’s the children who have to take care of the parents in terms of language. (P14)*

#### Feeling distrusted and misunderstood by staff in the healthcare

Respondents felt that the doctor and the registered nurses did not listen to them when they tried to communicate, regardless of whether an interpreter was present. Every time they got in contact with the healthcare centre, they could not get an appointment at all, or were told they had to wait or were in line for an appointment that they never got. They felt distrusted and expressed that they often met a new doctor and could not build a trusting relationship and had no continuity in care. Therefore, they tried to find the solution using “google”, which could end up with an adverse event due to wrong dosage:

*For example, I say that Sertraline is not good, that I can’t sleep when I take it. And then the doctor usually prescribes it again; so despite that, even though I’ve said it’s of no use, the doctor e prescribes the same thing. (P3)*

*I got very angry. I was really sick, and I needed to be on sick leave for a week or two, just to be able to get my energy back, to start to get better. But the doctor said “no, you have to learn the language”. Don’t you understand? I’m telling you I’m not well and I’m sick. It’s not that I don’t want to learn the language, but right now I’m not feeling well. And then I said, “when you can’t understand, I don’t want to come here. And I don’t even want to be treated”. (P3)*

### Independent and self-motivated

This category describes the experiences of the respondents who understood the information provided and felt that they could discuss different treatment alternatives with their doctor.

#### Managing to reach out and contact the healthcare centre

Some of the respondents experienced that they could manage the language well. They could call the healthcare centres, managed the answering machine with different choices, talked to the registered nurse who called back, and booked an appointment. Sometimes they had to call a few times before they could get an appointment; however, they were not dependent on another person to assist them. They felt trust in that they could get in contact with the healthcare centre if they, for example, needed to adjust the dosage of a medication:

*My doctor usually gives me an appointment every three or six months. But in the meantime, if something comes up and I need to contact the doctor, I have numbers for the counsellor and the coordinator. So I can contact them and they can talk to the doctor and arrange a doctor’s visit if necessary. (P22)*

#### Feeling understood and listened to

Some respondents felt that they could discuss with the doctor what medication they had, on what indications, as well as how and what time they should take them. They could also read and understand a medication list. During the observation of respondent 8: ‘The person shows all the boxes of medication, explains how to check the date of expiration on each box, and what time and on what indication to take the medication. After this, the person also shows the medication list and explains that this is where to double check all of the medications and if they don’t, or he/she doesn’t, understand the text, the doctor or the daughters make a translation so he/she can use it anyhow’.

The respondents felt trust in that they could manage either with an interpreter or without. They might want to have an interpreter if they saw a doctor, but not when visiting a physiotherapist; it was up to them to decide. Some of the respondents expressed that there were doctors or nurses who could speak their language at the healthcare centre. They felt trust that there was always someone who could give them information about the medication if they did not understand everything.

*If there is medicine where the prescription needs to be renewed, then the doctor does that. Like Gabapentin, in the beginning I had three, but when the doctor saw that I was getting worse and worse, the doctor increased the dose to three in the morning; the doctor listens. (P17)*

### Factors that can facilitate medication use

This category encompasses factors perceived to facilitate medication use, related to translation possibilities, and a sense that there was always a solution for receiving understandable information at the pharmacy.

#### Possibilities to translation

Most respondents did not have a wish or a suggestion about what could facilitate adherence to prescribed medication. However, when different suggestions were proposed, they were positive. Everyone expressed that an interpreter is the most important factor for facilitation. Almost all were positive about receiving a medication list in their own language. They expressed that it could facilitate their adherence to their medication, especially since many of them did not understand the information that the doctor gave them. During the observation of respondent 1: ‘The person had taken the insulin injection and was about to take the pills when reaching for the medication list in a plastic bag and gave it to the observer and expressed “I got that from the doctor, but I can’t read it so you can have it”. Another suggestion that the respondents were positive towards having was language selection on the telephone answering machine at the healthcare centre. The respondents felt that this service, available at e.g. the Swedish Social Insurance Agency and the Swedish Public Employment Service would make it easier for them to get in contact with the health care centre. If that opportunity were to be available, the participants would know that they could manage to reach out themselves and thus feel more secure:

*I can’t express myself in Swedish. At the Swedish Social Insurance Agency and the Swedish Public Employment Service, there, you can choose the language. But in healthcare, you can’t. That’s weird. (P17)*

#### There’s always a solution at the pharmacy to receive understandable information

Most of the respondents expressed that it was not possible to get an interpreter at the pharmacy. However, everyone was satisfied with the service at the pharmacy. They could usually find someone that could speak their language; also, many pharmacists were bilingual. Even if no one spoke their language, they always managed to explain in some way with hand signs or google translate. It did not matter if the person could not speak any Swedish at all. They always felt secure that they would receive information at the pharmacy. The respondents expressed that the pharmacist was very meticulous; often, they wrote information on the boxes in their language, such as the indication for the medication and what time to take the medication. During the observation of respondent 8: ‘The person shows the observer two of their medication boxes where the bilingual pharmacists have written the information in the respondent’s own language’:

*A person who works there, hi´s/she’s from X-land. So, when I collect the medicine, he/she helps me and writes, for example, like this. Hi/she wrote “I take it for high blood pressure”. So he/she helps. (P1)*

*When they talk and tell me, I understand quite well. So for me, it has never been a problem to get medicine or understand what they are saying at the pharmacy. I understand. (P22)*

## Discussion

This study is unique as it, through interviews and observations, explores diverse foreign-born people’s experiences and perceptions of self-management of medication, and if home-based practice patterns have implications on medication safety, and what factors may support safe medication. It adds new knowledge on migrants’ experiences of reaching their healthcare centre, facilitation in medication and communication at the pharmacy. The main findings showed that the two first categories represent different patterns among the respondents. One group where the respondents struggled and were dependent on another person to get information about medications, had difficulties in accessing the healthcare centre and felt that the staff did not trust them. The other group of respondents were independent and self-motivated in reaching out to the healthcare centre and in managing their medication. Having a medication list in the respondent’s own language and the option to choose their own language on the answering machine at the healthcare centre were perceived as factors that would facilitate their use/handling of medications. Although it was known that it was impossible to get an interpreter at the pharmacy, they felt there was always a solution for receiving understandable information, which made them feel safe. Notably, this study identified positive practices within pharmacies that contribute to preventing medication-related problems. The broader healthcare system can learn from these practices by incorporating workgroups that include multilingual and multicultural staff, providing lists with contact numbers for staff who speak different languages to facilitate communication and, most importantly, fostering a positive attitude towards patients and work tasks. These initiatives aim to find solutions and deliver high-quality services, thereby positively impacting medication safety for persons speaking different languages.

Is it possible that depending on the year and what kind of reception the respondents received when arriving in Sweden, affected their ability as migrants to get control over their own self-care and medication? In Sweden, the national law on activities for getting established for newly arrived migrants [24] was passed in 2010 under the Ministry of Employment. Newly settled migrants who have received a residence permit in Sweden are offered an Introduction programme, including a civic orientation course. This programme is intended to facilitate access to the labour market and promote integration. It has been found [25] that the Introduction programme provides an opportunity to develop knowledge and skills to gain more control over one’s life and participate in the Swedish society. However, this kind of programme has been different over the years in Sweden. Due to the high number of incoming refugees in 2015, referred to as the “refugee crisis” [26], it is not certain that everyone was offered the Introduction programme. Therefore, this could have affected the respondents’ opportunity to learn about the healthcare system. Thus, depending on what year they arrived in Sweden, this could have influenced their ability to gain control over their self-care and medication management. The findings of this study suggest that the establishment programme could be expanded to include more education in the Swedish language and information about the healthcare system, e.g. purchasing medications.

The majority of respondents in the present study experienced they could not reach/access the healthcare centre. Apart from language problems, it has been shown that lack of social support and other socioeconomic factors might also contribute to migrants having more difficulties in seeking care [4, 8–10]. An integrated care considers both lay and professional beliefs, aiming to provide individualised care that is thus, culturally congruent care [17]. The results of this study show that the healthcare centre is not completely accessible for this group of patients, namely migrants. Thus, the healthcare centre needs to be developed to be accessible for all patients, irrespective of background. As previously shown [17], the staff need to be trained to have adequate skills in identifying patients’ individual beliefs, practise and values. There is a need to design care in partnership between patients and staff. Together, they should develop an integrated care, including both professional and lay beliefs, practise and values to achieve the goal of good health and prevent illness. Therefore, the healthcare centre needs to develop and have a strategy/routine to identify these vulnerable patients to prevent adverse events and patient suffering.

This study indicates the need for implementing structures, such as staff education and assessments tools to assist healthcare providers in identifying patient’s needs and those who are vulnerable. Especially since health promotion of migrants in the early post-migration stage and communication is considered to be an important area for patient safety [27, 28]. Communication barriers or different cultural backgrounds increase the risks to patient safety [29]. The results show that there is a need to improve safety for migrant patients, and that this is still an under researched area as stated in a systematic review focusing on ethnic minority patients, although there were some migrants included [6].

With few exceptions, the majority of respondents in the present study claimed they fasted and changed their medication accordingly. This could be a medication safety risk and has been noted in diabetes care [30]. The recommendation is for the patient and the responsible physician to have a dialogue about suitability and risks that can arise from not taking the medication before the fasting begins. It is important to be aware of this and to understand that medication reviews can be a general way to avoid medication errors and to contribute to medication safety. Religious practices, such as fasting, can have a negative impact on health [30]. There may be differences between individual perceptions and the healthcare staff where the organisation’s rules and norms prevail [31, 32]. It is important to be observant of the risk of clashes in case of differing perceptions and, thus, always assess the individual’s perceptions.

The respondents in this study reported no or limited ability to speak Swedish, and one category that was identified was “dependent on another person ”. They experienced that they did not receive information about medication because of the language problems. Moreover, they had difficulties getting access to staff in healthcare and being able to communicate with them, and they felt distrusted and misunderstood. This is in line with previous studies [2–4], which have shown that it can be difficult for migrants to navigate and to access healthcare and that communication problems with staff at the healthcare centre can lead to inappropriate treatment. In fact, many adult migrants do not have appropriate health information and often face difficulties in managing health issues [33]. Good communication is essential to ensure patient safety, in terms of use of medication by patients [5].

A pictogram is a graphic symbol conveying specific meanings, which were not mentioned in our results. However, could their inclusion be a suggestion for situations where foreign-born do not understand the language? Previous use of pictograms has shown to contribute to patient safety/adherence if used in combination with good communication [34].

The findings in this study show that having an interpreter present is the best means to facilitate understanding and safe medication use. According to the Management Act [35], in Sweden, there is a right to use an interpreter in contact with all public authorities for persons who cannot speak Swedish, and the responsibility for calling upon the interpreter service and obtaining the provision of an interpreter lies with the healthcare service [36, 37]. However, there is a problem, since the pharmacies are not deemed to be a public authority and do not receive financial support for interpreters. Therefore, they do not use them. Good cooperation between healthcare and the interpreter service has been shown to contribute to high-quality healthcare for patients in need of interpreters to be able to communicate in healthcare [38]. A scoping review [39] shows that this is important for a relationship between the migrant and the healthcare professionals in order to promote adequate information about medications. A trusting relationship with the pharmacist leads to increased reliable information being shared about medications, a bridge to the cultural divide, and an understanding for their patient and, subsequently, their compassion and care being improved.

The findings in this study showed that half of the respondents received government support for their medication. Households in the Nordic countries are among the wealthiest in the world. However, in Sweden, the income inequality has increased at a fast pace during the past few decades. It is well established that economic inequality might lead to poverty and increased health inequalities [40]. In a systematic review [41], it is shown that socio-economic factors, including language proficiency, level of education, financial burdens, were common themes for non-adherence to medication, compromising patient safety. These findings might be a result of increasing inequalities.

### Limitations

We aimed to interview and observe all respondents in their homes; however, due to recruitment problem the majority, nine out of 15, were interviewed in their home and the rest in an environment that was considered familiar and a safe zone for the interviewees. Although patients were asked to participate at the healthcare centre, many turned down the offer. Certain groups of patients are difficult for researchers to access because of their social or physical location or vulnerability. This group of patients are vulnerable, not least due to their experiences related to migration (most were refugees), and therefore, deemed challenging to recruit for research [42]. While the observation contexts differed, the impact is considered negligible as the same data collection instruments/methods were used, with only storage and home environment differing in observations made at the voluntary organisation, where the majority were conducted at home.

The use of an interpreter was essential for communicating with respondents. To reduce the influence of interpreters, we minimised the number of interpreters and only used gender-matched professionally authorised persons [43].

The purposeful sampling strategy aimed to include information-rich cases to deepen our understanding of the studied area [18] and, therefore, explanations cannot be draw from it.

All but one of the respondents were females, which is a limitation. It cannot be ruled out that the results could have been different with an increased number of male respondents. However, carefully collected, analysed and described results are transferable to groups and contexts similar in characteristics [18].

Potential limitations related to the sampling procedure that could affect the results within the studied group, e.g. education level and time of residency in Sweden, may introduce sampling bias. However, this was considered in the analysis, and no such differences were found regarding educational level or time of residency in Sweden within sub-groups. However, due to the qualitative study design, such relationships cannot be excluded. The study design made it possible to explore different perceptions and experiences to get a deeper understanding of the studied phenomenon, albeit not to seek explanations.

### Relevance for clinical practice

The study highlights the importance of identifying individual experiences and perceptions of self-management and medication to visualise aspects that can be improved to promote health and well-being.

## Conclusion

In conclusion, this exploratory study highlights that language barriers in healthcare can affect medication safety. Two groups with different patterns were identified: one that struggled and was always dependent on another person to access and communicate with staff in the healthcare centre. The other group struggled too, but they were able to get access to and communicate with the healthcare staff on their own. Being aware of migrants with communication barriers is essential to design and implement future facilitation of medication. Thus, having a medication list in the patient’s own language or the option to choose one’s language on the answering machine in clinical practice could improve the communication between the patient and the staff. Furthermore, it could lead to culturally congruent care based on the individual’s beliefs, practise and values However, many of the respondents felt that, at the pharmacy, there was always a solution for receiving information about medications that was understandable. This new knowledge on migrants’ experiences of being able to access the healthcare centre, facilitation in medication and communication at the pharmacy should be used to understand the gap in communication barriers in order to increase patient safety. It is important to promote equality. The findings in this study show one picture of a problem, and it is important to explore the problem from other angles, e.g. perspectives from staff in pharmacies/healthcare as well as inputs from relatives. Therefore, further studies are needed in this area.

## Data Availability

The datasets generated and/or analysed during the current study are not publicly available in order to protect the integrity, anonymity, and confidentiality of the respondents, with the exception that anonymised quotations supporting the data in the results section in the manuscript are shown. These are available from the corresponding author upon reasonable request.
